# No evidence of impediment by three common classes of prescription drugs to post-stroke aphasia recovery in a retrospective longitudinal sample

**DOI:** 10.1371/journal.pone.0270135

**Published:** 2022-06-24

**Authors:** Melissa D. Stockbridge, Zafer Keser, Lisa D. Bunker, Argye E. Hillis

**Affiliations:** 1 Department of Neurology, Johns Hopkins University School of Medicine, Baltimore, Maryland, United States of America; 2 Department of Neurology, Mayo Clinic, Rochester, Minnesota, United States of America; 3 Department of Physical Medicine and Rehabilitation, Johns Hopkins University School of Medicine, Baltimore, Maryland, United States of America; 4 Department of Cognitive Science, Krieger School of Arts and Sciences, Johns Hopkins University, Baltimore, Maryland, United States of America; The University of Edinburgh, UNITED KINGDOM

## Abstract

A number of pharmaceuticals have been identified as potential adjuvants to speech language therapy following stroke, but it is also important to consider which pharmaceuticals may result in a *less* robust recovery. Here we examine whether post-stroke language recovery was meaningfully impeded by cholinergic, GABAergic, or dopaminergic medications patients received. Eighty participants with left hemisphere stroke were examined retrospectively to see whether the use of one of these three classes of medication prior to admission for acute stroke, during their inpatient stay, or at discharge was associated with differences in recovery on three common measures of language. While prescription of any of the candidate drugs was relatively uncommon, groups were very well matched for many common factors that impact performance. When age, education, and acute lesion volume were controlled, there were no significant differences in performance among those taking cholinergic, GABAergic, or dopaminergic medications and those who were not. Those who experienced a “good recovery” of language (≥10% improvement on any one language measure over time) had similar exposure to these drugs to those with a poor recovery. This work represents a first look at these drug classes with regard to their effects on the recovery of language after stroke and should not be interpreted as resolving all potential for concern, but these results do offer modest reassurance that these common classes of pharmacotherapy, when given for short periods in this population, do not appear to have marked deleterious effects on post-stroke recovery of language.

## Introduction

Speech and language therapy is the standard treatment of post-stroke aphasia [1]. A number of pharmaceuticals have been identified as potential adjuvants to speech language therapy, leading to numerous active lines of research [see 2, for review, 3]. Most notably, there is evidence that cholinergic [NCT04134416; 4–6], GABAergic [NCT00227461; 7–11], and serotonergic mechanisms [NCT03843463; 12–14] may facilitate plasticity-dependent recovery of language. Given such findings, it is at least as important to consider underlying mechanisms of pharmaceuticals that may result in a *less* robust recovery from stroke.

Barbiturates [15–18], alpha blockers [19], benzodiazepines [20], dopamine antagonists [21–23], and norepinephrine-dopamine reuptake inhibitors [20] all have demonstrated deleterious effects on post-stroke recovery. Moreover, recent work has sparked enthusiastic investigation of cholinergic antagonists for their contribution to cognitive decline when taken long term [24–28]. This research has been spurred in part by the mild beneficial effect of cholinesterase inhibitors on reducing the rate of cognitive decline in Alzheimer’s disease.

These concurrent observations of pharmaceutical activity associated with recovery along with the potential for detrimental cognitive effects of other medications has led to the present investigation. Here we examine whether post-stroke recovery in our patients with left hemisphere strike was meaningfully impeded by medications they received to address co-morbid diagnoses or manage symptoms. Three candidate mechanisms were examined in our longitudinal retrospective cohort of patients with left-hemisphere stroke: cholinergic (e.g., competitive nicotinic acetylcholine (ACh) receptor antagonists, "reversible" carbamate inhibitors, muscarinic receptor antagonists), GABAergic (e.g., benzodiazepines, barbiturates), and dopaminergic (e.g., DAT ligands, 5-HT_2A_ and D_2_ antagonists, triptans).

## Methods

### Records reviewed

A total of 80 participants with left hemisphere ischemic lesions were identified through an ongoing longitudinal protocol investigating stroke recovery that were approved by the Johns Hopkins University School of Medicine Institutional Review Board. All participants provided written consent and were 18+ year-old proficient English speakers with normal or corrected-to-normal vision and hearing and did not demonstrate history of neurologic conditions affecting the brain other than stroke. In order to be included in this analysis, participants had to have been evaluated during the acute phase of recovery, and then again during the chronic phase of recovery, using one or more of three classic outcome measures of aphasia: the aphasia quotient (AQ, i.e., severity) from the Western Aphasia Battery [WAB, an omnibus measure of language; 29], the Boston Naming Test (BNT; picture naming of nouns), and the amount of previously identified and normed content (“content units”; CUs) provided when describing the *Cookie Theft* picture from the Boston Diagnostic Aphasia Examination [30].

The *acute phase* of recovery is defined as within the first week following stroke, often the same day as admission (Mean = 3 days after stroke ± 3 days). The *chronic phase* of recovery is defined as at least 6 months after stroke. Per the longitudinal protocol, patients were contacted for follow up during the chronic phase at 6 and 12 months after stroke; although, a small number were evaluated two or more years after stroke (N = 9; 11%). Performance during the chronic period generally has stabilized; thus, if a participant was seen for more than one evaluation after 6 months, their performance across these timepoints was averaged. Change was then quantified by calculating the difference in performance scores between the acute and chronic phases, divided by the acute score to normalize the change regardless of initial acute severity. Where acute scores were 0, an artificial minimal score of 1 on the WAB was used to calculate the corrected improvement.

During the period of longitudinal data collection, the classic *Cookie Theft* image was replaced with a more contemporary, color image [31]. 42 patients described only the classic *Cookie Theft* image, 12 patients described only the new image, and 10 patients described both. These images are *not* interchangeable, with the updated version containing significantly greater total content. However, change in CU divided by baseline CU is comparable, and was statistically similar in this sample (Old: Mean change in content units = 2 units, SD = 4; New: Mean = 2, SD = 4 units; t(62) = 0.10, p = 0.91). Therefore, in order to maximize sample size, performance on either the old or new *Cookie Theft* was used and, if both pictures were administered, the change in each was calculated separately and then averaged.

### Prescription records

Prescription records were obtained from the electronic medical records at three distinct time points. Medications prior to stroke were deduced from initial neurology history and physical notes at admission and cross referenced with discharge summary notes, as many patients are unable to communicate medical history at admission but later may improve or have family who provide additional history. Scheduled inpatient medications were found in the electronic Medication Administration Record (MAR). For 26 patients (those whose hospitalization was before January 2015), these records could not be accessed due to a change in the underlying electronic record system. Discharge medications were found in the discharge summaries. Though we recognize the possibility that discharged patients could choose not to comply with prescriptions, it was assumed that prescribed medications were used in the intervening months between hospitalization and follow-up. Prescription drug lists for candidate mechanisms are shown in S1 File. Drug list, informed by Goodman & Gilman’s [32], DailyMed (https://dailymed.nlm.nih.gov/dailymed/), and U.S. Food and Drug Administration approval history. There were 153 drugs identified.

### Lesion/infarct segmentation

Acute ischemic lesions were delineated and quantified manually by researchers and clinicians experienced in lesion segmentation using standard of care diffusion weighted images (DWI; N = 75). Trained study team members manually traced lesions on DWI scans using MRIcron or MRIcroGL (available at nitrc.org). Tracings then were verified by experienced researchers. We used routines from SPM12 (Statistical Parameter Mapping; https://www.fil.ion.ucl.ac.uk/spm/software/spm12/) to warp each patient’s DWI b0 image to a healthy older adult template [33] and subsequently applied the normalization parameters to the lesion map. We calculated the volume of the normalized lesion map (in mm3) using NiiStat (https://www.nitrc.org/projects/niistat/).

### Statistical analysis

Two complementary analyses were planned. First, a multivariable analysis of variance was conducted in which the Western Aphasia Battery AQ, Boston Naming Test, and *Cookie Theft* content units were dependent variables and whether a cholinergic, GABAergic, or dopaminergic drug was administered at any point (i.e., collapsing across pre-stroke, scheduled during hospitalization, and discharge time points) were fixed factors (α = 0.05/3 = 0.017). Given the small samples available for each permutation of mechanism and time point, we decided to collapse across timepoints for a given drug mechanism.

In a complementary analysis done to maximize the analyzable data, standardized differences in WAB-AQ, BNT, and CU were dichotomized into individuals with 10% or greater improvement on any of the three measures (“good recovery”) and those who did not show at least 10% improvement on any of the three measures (“poor recovery”). This allowed for much more similar group numbers for comparison. Recovery groups then were examined for the likelihood of taking a drug with a given mechanism. Data are available in S2 File.

## Results

### Description of groups

**Fig 1:**
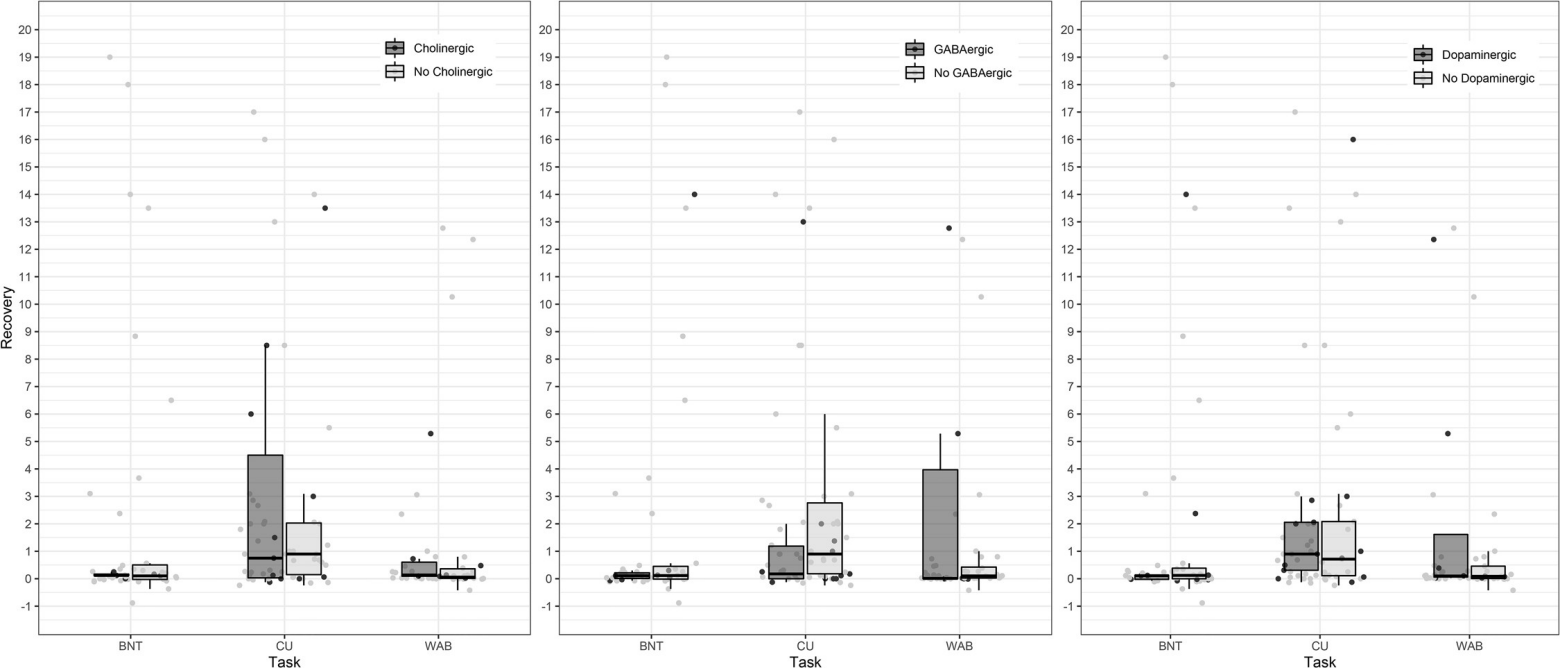
Change in performance by group. Scores in the table reflect standardized difference scores between acute performance and average chronic performance. In each case, the drug group is represented by the darker grey, while the no drug group is represented in lighter grey. Data points are jittered slightly on the x-axis to increase visibility of overlapping scores (within task x-axis differences are not meaningful). The outlier patient previously described with an improvement in WAB AQ of 51.5 was not included in the figure.

Overall, prescription of any of the candidate drugs was relatively uncommon in our sample and included only 26 of the 153 identified products (Table 1). Despite a fairly large number of records available, only 13 individuals had been on a cholinergic at any time, 14 had been on a GABAergic medication, and 16 had been on a dopaminergic medication. A total of 31 individuals had been prescribed any of the three at any time point examined. One patient, a 62-year-old male who received both a GABAergic and dopaminergic scheduled during admission experienced a recovery on the WAB from an AQ of 0.3 (global aphasia) to an AQ of 51.5 at 6 months post-stroke (standardized improvement of 171.67). In order to utilize this patient in analysis, an artificial minimal score of 1 on the WAB was used to calculate the corrected improvement. This outlier patient was not included in Fig 1.

**Table 1:** N prescriptions by drug class.

Drug	Admission	Inpatient	Discharge	Total
**Cholinergic**	6	8	9	23
Nicotine	1	5	3	9
Ipratropium	1	1	2	4
Cyclobenzaprine	2	0	1	3
Carisoprodol	1	1	0	2
Dicyclomine	1	0	1	2
Scopolamine	0	1	0	1
Solifenacin	0	0	1	1
Tiotropium	0	0	1	1
**GABAergic**	**4**	**15**	**3**	**22**
Lorazepam	0	8	0	8
Alprazolam	1	1	0	2
Midazolam	0	2	0	2
Valproate	0	1	0	1
Zolpidem	1	0	0	1
Gabapentin	2	2	3	7
Zonisamide	0	1	0	1
**Dopaminergic**	**13**	**9**	**10**	**32**
Pramipexole	2	1	2	5
Quetiapine	2	1	2	5
Trazodone	1	2	2	5
Bupropion	1	1	2	4
Duloxetine	1	1	1	3
Ondansetron	1	2	0	3
Amphetamine	1	0	1	2
Asenapine	1	1	0	2
Methylphenidate	1	0	0	1
Nortriptyline	1	0	0	1
Sumatriptan	1	0	0	1

N refers to number of prescriptions summed across timepoints, not number of people with a prescription, and were not necessarily mutually exclusive cases.

#### Cholinergic

Individuals who had received a cholinergic prescription were age-matched to individuals who did not receive a cholinergic (t(77) = 1.29, p = 0.20). They had similar levels of education (t(75) = 1.71, p = 0.09). Where available (N = 75) acute lesion volume (t(73) = 1.75, p = 0.08) between the two groups was considered and also found not to be significantly different between groups. There were no differences based on Fisher’s exact tests of sex (p = 0.11), handedness (p = 1.0), likelihood of having diabetes (DM; p = 1.0), hypertension (HTN; p = 1.0), or psychiatric diagnoses (Psy; p = 1.0). Groups also were matched on acute aphasia severity as measured by WAB AQ (Drug: 66.3±37.7, No Drug: 75.4±30.0, t(51) = 0.79, p = 0.43).

#### GABAergic

Those taking a GABAergic prescription tended to be younger at the time of their stroke (t(77) = 2.76, p = 0.007). There were no differences in sex (p = 0.37), handedness (p = 0.42), DM (p = 1.0), HTN (p = 0.63), or psychiatric diagnoses (p = 0.61), education (t(75) = 0.70, p = 0.48), or lesion volume (t(73) = 0.56, p = 0.58). Groups also were matched on acute aphasia severity as measured by WAB AQ (Drug: 68.5±44.3, No Drug: 75.3±27.3, t(51) = 0.63, p = 0.53).

#### Dopaminergic

Individuals with and without dopaminergic prescriptions were matched in all dimensions examined: age (t(77) = 1.87, p = 0.07), education (t(75) = 0.18, p = 0.86), lesion volume (Levene’s F = 2.5, p = 0.037, corrected t(13.68) = 0.93, p = 0.37), sex (p = 0.83), handedness (p = 0.47), DM (p = 1.0), HTN (p = 0.35), and Psy (p = 0.18). Groups also were matched on acute aphasia severity as measured by WAB AQ (Drug: 65.9±40.1, No Drug: 76.2±28.2, t(51) = 1.01, p = 0.32).

### Multivariate analysis of variance

Improvement on each measure by individuals in each drug group is summarized in Table 2. When age, education, and acute lesion volume were controlled, Pillai’s trace was not significant for any substance: cholinergic (V = 0.07; F(3, 10) = 0.23, p = 0.87), GABAergic (V = 0.04; F(3, 10) = 0.13, p = 0.94) or dopaminergic (V = 0.02; F(3, 10) = 0.06, p = 0.98). The assumption of homogeneity of variances was upheld for all contrasts.

**Table 2:** Demographic and performance summary by mechanism.

	M:F	Age	Edu	Hand	Vol	DM	HTN	Psy	WAB	BNT	CU
Cholinergic	7:5	12	13	12;1	12	4;8	11;1	1;11	7	5	11
		56.5±13.90	13.08±2.14		51475±74118				0.98±1.92	0.13±0.09	3.03±4.49
No Cholinergic	30:37	67	64	58;7	63	23;44	60;7	7;59	39	40	43
		61.5±12.09	14.51±2.85		24111±43950				2.49±8.66	2.27±5.09	2.47±4.4
GABAergic	9:5	14	13	12;1	14	5;9	12;2	2;11	7	7	11
		52.79±14.05	13.77±2.35		38049±75911				9.94±18.94	2.07±5.26	1.62±3.84
No GABAergic	28:37	65	64	58;7	61	22;43	59;6	6;59	39	38	43
		62.45±11.44	14.37±2.87		26296±43060				0.88±2.55	2.02±4.83	2.83±4.51
Dopaminergic	8:8	16	14	13;2	13	5;11	13;3	3;12	9	9	13
		55.63±13.68	14.14±2.25		45146±75961				7.76±16.92	1.84±4.63	2.25±4.26
No Dopaminergic	29:34	63	63	57;6	62	22;41	58;5	5;58	37	36	41
		62.03±11.83	14.29±2.90		24997±43256				0.92±2.67	2.07±4.95	2.68±4.46

Continuous variables are reported as N Mean ± Standard Deviation. Edu: Education (in years). Hand: Handedness right: left. Vol: Acute lesion volume (mm^3^). DM: Diabetes mellitus present;absent. HTN: hypertension present;absent. Psy: mental health diagnosis in chart at the time of admission present;absent. For assessments, scores in the table reflect standardized difference scores between acute performance and average chronic performance. Higher numbers are associated with greater recovery. WAB: Western Aphasia Battery Aphasia Quotient (omnibus measure of language). BNT: Boston Naming Test (picture naming of nouns). CU: *Cookie Theft* content units (content provided when describing a picture).

As shown in Fig 1, there was a large range in recovery scores for each test, for patients with each of the potentially detrimental medications. Although there might appear to exist a slight trend for patients without GABAergic drugs to show more improvement in CU and less improvement on the WAB, the opposite directions of the trends make it likely that these trends are not reliable or meaningful. As indicated by the individual data points, these trends are probably due to 1–2 outliers, like the apparent trend for patients on cholinergic antagonists to show more improvement in CU.

#### Likelihood of good recovery

Of the 80 patients examined, half were identified as having made a good recovery (≥ 10% improvement on any of the three language measures examined). Recovery groups were matched in age at the time of stroke (t(77) = 1.02, p = 0.31), education (t(75) = 0.71, p = 0.48), lesion volume (t(73) = 1.1, p = 0.27), sex (p = 0.82), handedness (p = 0.10); DM (p = 1.0); HTN (p = 1.0), and likelihood of a mental health diagnosis (p = 0.26; Table 3). Individuals who experienced a good recovery had nearly identical likelihoods to those with poor recovery of have been taking a cholinergic (p = 1.0), GABAergic (p = 0.77), or dopaminergic (0.40) drug (Table 4). When explored, pre-admission, scheduled inpatient, and discharge timepoints considered separately were not associated with any significant differences.

**Table 3:** Demographic and performance summary by recovery group.

	M:F	Age	Edu	Hand	Vol	DM	HTN	Psy	WAB	BNT*	CU*
Good recovery	19:20	39	37	32; 6	37	13;26	35;4	2;36	19	20	27
		62.2±12.74	14.5±2.93		34983±42770				5.16±11.98	4.61±6.44	4.80±5.34
Poor recovery	18:22	40	40	38;2	38	14;26	36;4	6;34	27	25	27
		59.33±12.07	14.05±2.65		22167±56665				0.22±1.02	-0.03±0.21	0.36±0.54

*p < 0.01. Continuous variables are reported as N Mean ± Standard Deviation. Edu: Education (in years). Hand: Handedness right; left. Vol: Acute lesion volume (mm^3^). DM: Diabetes mellitus present; absent. HTN: hypertension present; absent. Psy: mental health diagnosis in chart at the time of admission present; absent. For assessments, scores in the table reflect standardized difference scores between acute performance and average chronic performance. Higher numbers are associated with greater recovery. WAB: Western Aphasia Battery Aphasia Quotient (omnibus measure of language). BNT: Boston Naming Test (picture naming of nouns). CU: *Cookie Theft* content units (content provided when describing a picture).

**Table 4:** Patient grouping by recovery group and drug mechanism.

	Good	Poor
	N = 40	N = 40
Cholinergic	6	7
No Cholinergic	34	33
GABAergic	6	8
No GABAergic	34	32
Dopaminergic	6	10
No Dopaminergic	34	30

## Discussion

In this investigation, we examined whether three candidate drug classes with the mechanistic potential to interrupt or impede neuroplasticity were associated with measurable differences in language recovery after stroke, as measured using an omnibus language assessment (WAB), picture naming task (BNT), and picture description task (*Cookie Theft*). When controlling for other important group differences in demographic factors and health status, neither a general linear model-based analysis nor an analysis of the contingency tables produced evidence that there was a detrimental effect (or, indeed, *any* effect) of cholinergic, GABAergic, or dopaminergic prescriptions on language recovery. Overall, there were relatively few individuals within our sample who had been prescribed the drugs under consideration whether prior to admission, during hospitalization, or at discharge. This low signal within our sample led us to adopt a multi-pronged statistical approach.

Recently there has been more interest in studying potential beneficial pharmaceuticals such as serotonergic drugs [12] and neuro-stimulants [34, 35] in a controlled clinical trial setting to promote post-stroke recovery. It is equally crucial to avoid medications that may interfere with the post-stroke recovery in accordance with the “first, do no harm” principle. However, it is unethical to design a research study in which potentially harmful medications are tested directly. Thus, despite the inherent limitations and biases of retrospective analysis, these studies play a crucial role in guiding treating clinicians in their therapeutic armamentarium when dealing with various post-stroke impairments and complications in a balanced fashion.

Although the goal of the study was not to study drug prescription patterns after stroke, we observed that GABAergic medications were prescribed more commonly for younger patients. We speculate that the treating clinicians might have liberally chosen pharmacotherapy over other methods (e.g., physical restraint) to control post-stroke agitation and/or anxiety. Larger studies matching clinical indications for GABAergic medications are needed to further investigate prescription patterns and their potential effect on post-stroke recovery in different age groups. It also is interesting to note that certain drugs perceived to enhance cognitive performance were not readily prescribed in our sample despite recognized safety and efficacy profiles in the acute stroke period [36, 37]. This reinforces the notion that continuing research on the pharmacotherapy of post-stroke aphasia is imperative to gain further knowledge on the detrimental and beneficial drugs for poststroke aphasia and eventually translating research findings to clinical practice [38]. Important initiatives in this direction are ongoing [39, 40].

This work represents a first look at these drug classes with regard to their effects on the recovery of language after stroke and should not be interpreted as resolving all potential for concern. There are some important limitations of this work that temper our ability to generalize from these findings. First, these analyses are likely underpowered. Relatively few individuals in our sample had any record of having taken any of the drugs we examined. Nevertheless, there were no strong trends that would suggest that a significant effect would emerge with greater power. Second, there were a number of potential sources of variability we were unable to control due to small sample size. We did not examine patients’ over-the-counter drug or substance use, nor did we have the information with which to ensure patients had complied with drugs prescribed at admission or discharge. We have not included drug route of administration and dosages in the analysis, and we were forced to collapse across time points we did have (admission versus inpatient versus discharge) in order to maximize the usable data. It seems plausible that effect sizes are sufficiently small that only over long-term/life-long exposure are candidate mechanisms worthy of concern and that the window in which we examined our patients–from admission to discharge–was too narrow for an effect to be seen. Finally, certain information about these patients was unavailable, namely the administration of hyperacute rTPA and the availability or duration of behavioral speech-language therapy. We feel it is safe to presume that rTPA would have been used if consented to among patients for whom it was appropriate and that the amount, character, and duration of speech therapy was provided as clinically indicated.

Nevertheless, these results offer modest reassurance that these common classes of pharmacotherapy, when given for short periods in this population do not appear to have marked deleterious effects on post-stroke recovery of language. Additional prospective analyses with greater specificity with regard to mechanism and duration are needed to fully appreciate the relationship these drugs may play in plasticity-dependent recovery of language in the post-stroke period.

## Supporting information

S1 File Drug list. (DOCX)

S2 File Data set. (XLSX)
