# Supplementary material for: No evidence of impediment by three common classes of prescription drugs to post-stroke aphasia recovery in a retrospective longitudinal sample
Source: PLoS One. 2022 Jun 24;17(6):e0270135. doi: 10.1371/journal.pone.0270135 (PMC9231759; doi:10.1371/journal.pone.0270135)
Supplement: S1 File — (DOCX) [file pone.0270135.s001.docx]

| **Supporting Information A: Drug candidate list based on Goodman & Gillman (2017)** | | | |
| --- | --- | --- | --- |
| **Drug** | **Category** | **Mechanism** | **Common Use*** |
| Ambenonium | Cholinergic | "reversible" carbamate inhibitor | Myasthenia gravis |
| Neostigmine | Cholinergic | "reversible" carbamate inhibitor | Myasthenia gravis; Paralytic ileus and atony of the urinary bladder |
| Pyridostigmine | Cholinergic | "reversible" carbamate inhibitor | Myasthenia gravis |
| Rivastigmine | Cholinergic | "reversible" carbamate inhibitor, acetylcholinesterase inhibitor | Memory loss in Alzheimer’s and Parkinson’s disease |
| Abobotulinumtoxin A | Cholinergic | agent that blocks ACh release | Cervical dystonia; Glabellar lines |
| Incobotulinumtoxin A | Cholinergic | agent that blocks ACh release | Blepharospasm; Cervical dystonia; Glabellar lines |
| Onabotulinumtoxin A | Cholinergic | agent that blocks ACh release | Axillary hyperhidrosis (severe), Blepharospasm associated with dystonia; Cervical dystonia; Migraine (chronic) prophylaxis; Overactive bladder; Strabismus; Upper limb spasticity (severe); Urinary incontinence (due to detrusor overactivity associated with a neurologic condition) |
| Rimabotulinumtoxin B | Cholinergic | agent that blocks ACh release | Cervical dystonia |
| Carisoprodol | Cholinergic | CNS-active agent | Muscle relaxants |
| Cyclobenzaprine | Cholinergic | CNS-active agent | Muscle spasm |
| Metaxalone | Cholinergic | CNS-active agent | Muscle relaxants |
| Methocarbamol | Cholinergic | CNS-active agent | Muscle relaxants |
| Orphenadrine | Cholinergic | CNS-active agent | Muscle relaxants |
| Atracurium | Cholinergic | competitive nicotinic ACh receptor antagonist | Renal failure |
| Cisatracurium | Cholinergic | competitive nicotinic ACh receptor antagonist | Renal failure |
| Doxacurium | Cholinergic | competitive nicotinic ACh receptor antagonist | Renal failure |
| Gantacurium | Cholinergic | competitive nicotinic ACh receptor antagonist | Renal failure |
| Metocurine | Cholinergic | competitive nicotinic ACh receptor antagonist | Surgery, Paralytic agent |
| Mivacurium | Cholinergic | competitive nicotinic ACh receptor antagonist | Surgery, Paralytic agent |
| Pancuronium | Cholinergic | competitive nicotinic ACh receptor antagonist | Surgery, Paralytic agent |
| Pipecuronium | Cholinergic | competitive nicotinic ACh receptor antagonist | Renal failure |
| Rocuronium | Cholinergic | competitive nicotinic ACh receptor antagonist | Surgery, Paralytic agent |
| Vecuronium | Cholinergic | competitive nicotinic ACh receptor antagonist | Surgery, Paralytic agent |
| Memantine | Cholinergic (Indirect)  Glutamatergic (Direct) | low-affinity uncompetitive NMDA antagonist | Alzheimer’s disease |
| Benztropine | Cholinergic | mixed mechanism of action - muscarinic receptor antagonist | Parkinson’s disease |
| Trihexyphenidyl | Cholinergic | mixed mechanism of action - muscarinic receptor antagonist | Parkinson’s disease |
| Bethanechol | Cholinergic | muscarinic receptor agonist | Urinary retention, Ileus |
| Cevimeline | Cholinergic | muscarinic receptor agonist | Xerostomia due to Sjögren syndrome |
| Aclidinium | Cholinergic | muscarinic receptor antagonist | Chronic obstructive pulmonary disease; Rhinorrhea |
| Atropine | Cholinergic | muscarinic receptor antagonist | Acute symptomatic bradycardia; Cholinesterase inhibitor intoxication; Aspiration prophylaxis |
| Darifenacin | Cholinergic | muscarinic receptor antagonist | Overactive bladder; Enuresis; Neurogenic bladder |
| Dicyclomine | Cholinergic | muscarinic receptor antagonist | Irritable bowel syndrome |
| Fesoterodine | Cholinergic | muscarinic receptor antagonist | Overactive bladder; Enuresis; Neurogenic bladder |
| Hyoscyamine | Cholinergic | muscarinic receptor antagonist | Irritable bowel syndrome |
| Ipratropium | Cholinergic | muscarinic receptor antagonist | Chronic obstructive pulmonary disease; Rhinorrhea |
| Oxybutynin | Cholinergic | muscarinic receptor antagonist | Overactive bladder; Enuresis; Neurogenic bladder |
| Scopolamine | Cholinergic | muscarinic receptor antagonist | Motion sickness |
| Solifenacin | Cholinergic | muscarinic receptor antagonist | Overactive bladder; Enuresis; Neurogenic bladder |
| Tiotropium | Cholinergic | muscarinic receptor antagonist | Chronic obstructive pulmonary disease; Rhinorrhea |
| Tolterodine | Cholinergic | muscarinic receptor antagonist | Overactive bladder; Enuresis; Neurogenic bladder |
| Trospium | Cholinergic | muscarinic receptor antagonist | Overactive bladder; Enuresis; Neurogenic bladder |
| Umeclidinium | Cholinergic | muscarinic receptor antagonist | Chronic obstructive pulmonary disease; Rhinorrhea |
| Nicotine | Cholinergic | nicotinic ACh receptor agonist | Smoking, Smoking cessation |
| Succinylcholine | Cholinergic | nicotinic ACh receptor agonist | Surgery, skeletal muscle relaxation |
| Varenicline | Cholinergic | nicotinic ACh receptor agonist | Smoking cessation |
| Donepezil | Cholinergic | noncovalent reversible inhibitor, acetylcholinesterase inhibitor | Alzheimer’s disease |
| Galantamine | Cholinergic | noncovalent reversible inhibitor, acetylcholinesterase inhibitor | Alzheimer’s disease |
| Tacrine | Cholinergic | noncovalent reversible inhibitors | Alzheimer’s disease |
| Alprazolam | GABAergic | Benzodiazepines dynergistic with other CNS depressants | Anxiety disorders |
| Chlordiazepoxide | GABAergic | Benzodiazepines dynergistic with other CNS depressants | Anxiety disorders; Alcohol withdrawal |
| Clorazepate | GABAergic | Benzodiazepines dynergistic with other CNS depressants | Anxiety disorders |
| Estazolam | GABAergic | Benzodiazepines dynergistic with other CNS depressants | Anxiety disorders |
| Flurazepam | GABAergic | Benzodiazepines dynergistic with other CNS depressants | Insomnia |
| Lorazepam | GABAergic | Benzodiazepines dynergistic with other CNS depressants | Anxiety disorders; Alcohol withdrawal,  Antiseizure |
| Midazolam | GABAergic | Benzodiazepines dynergistic with other CNS depressants | Intraoperative medication; Preanesthetic  Antiseizure |
| Oxazepam | GABAergic | Benzodiazepines dynergistic with other CNS depressants | Anxiety disorders; Alcohol withdrawal |
| Quazepam | GABAergic | Benzodiazepines dynergistic with other CNS depressants | Insomnia |
| Temazepam | GABAergic | Benzodiazepines dynergistic with other CNS depressants | Insomnia |
| Triazolam | GABAergic | Benzodiazepines dynergistic with other CNS depressants | Insomnia |
| Stiripentol | GABAergic | GABA uptake/GABA transminase inhibitor | Antiseizure |
| Tiagabine | GABAergic | GABA uptake/GABA transminase inhibitor | Antiseizure |
| Vigabatrin | GABAergic | GABA uptake/GABA transminase inhibitor | Antiseizure |
| Clobazam | GABAergic | GABA_A_ receptor allosteric modulator (benzodiazepines, barbituates) | Antiseizure |
| Clonazepam | GABAergic | GABA_A_ receptor allosteric modulator (benzodiazepines, barbituates) | Antiseizure |
| Diazepam | GABAergic | GABA_A_ receptor allosteric modulator (benzodiazepines, barbituates) | Antiseizure |
| Phenobarbital | GABAergic | GABA_A_ receptor allosteric modulator (benzodiazepines, barbituates) | Antiseizure |
| Primidone | GABAergic | GABA_A_ receptor allosteric modulator (benzodiazepines, barbituates) | Antiseizure  Essential tremor |
| Valproate | GABAergic | mood stabilizer, mixed mechanism of action | Bipolar disorder, Antiseizure  Headache |
| Eszopiclone | GABAergic | Z compounds - non-benzodiazepines with agonist effects at GABA_A_ receptors | Insomnia |
| Zaleplon | GABAergic | Z compounds - non-benzodiazepines with agonist effects at GABA_A_ receptors | Insomnia |
| Zolpidem | GABAergic | Z compounds - non-benzodiazepines with agonist effects at GABA_A_ receptors | Insomnia |
| Gabapentin | GABAergic | Alpha-2-delta calcium channel ligand | Antiseizure  Neuropathic pain |
| Pregabalin | GABAergic | Alpha-2-delta calcium channel ligand | Antiseizure  Neuropathic pain |
| Ethosuximide | GABAergic | blocks alpha 1 subunit of T-type calcium channel (thalamic action potentials) | Antiseizure |
| Zonisamide | GABAergic | blocks alpha 1 subunit of T-type calcium channel (thalamic action potentials) | Antiseizure |
| Amantadine | GABAergic | mixed mechanism of action | Parkinson’s disease |
| Aripiprazole | Dopaminergic | D_2_ partial agonist, atypical antipsychotic | Major depressive disorder; Psychotic disorders; bipolar disorder; Schizophrenia |
| Almotriptan | Dopaminergic | 5-HT_1B/1D_ receptor agonists (triptans) | Migraine-abortive |
| Eletriptan | Dopaminergic | 5-HT_1B/1D_ receptor agonists (triptans) | Migraine-abortive |
| Frovatriptan | Dopaminergic | 5-HT_1B/1D_ receptor agonists (triptans) | Migraine-abortive |
| Naratriptan | Dopaminergic | 5-HT_1B/1D_ receptor agonists (triptans) | Migraine-abortive |
| Rizatriptan | Dopaminergic | 5-HT_1B/1D_ receptor agonists (triptans) | Migraine-abortive |
| Sumatriptan | Dopaminergic | 5-HT_1B/1D_ receptor agonists (triptans) | Migraine-abortive |
| Zolmitriptan | Dopaminergic | 5-HT_1B/1D_ receptor agonists (triptans) | Migraine-abortive |
| Asenapine | Dopaminergic | 5-HT_2A_ and D_2_ antagonist | Schizophrenia |
| Iloperidone | Dopaminergic | 5-HT_2A_ and D_2_ antagonist | Schizophrenia |
| Paliperidone | Dopaminergic | 5-HT_2A_ and D_2_ antagonist | Schizophrenia |
| Sertindole | Dopaminergic | 5-HT_2A_ and D_2_ antagonist | Schizophrenia |
| Ziprasidone | Dopaminergic | 5-HT_2A_ and D_2_ antagonist | Schizophrenia |
| Lurasidone | Dopaminergic | 5-HT_2A_ and D_2_ antagonist, atypical antipsychotic | Major depressive disorder; Psychotic disorders; bipolar disorder; Schizophrenia |
| Olanzapine | Dopaminergic | 5-HT_2A_ and D_2_ antagonist, atypical antipsychotic | Major depressive disorder; Psychotic disorders; bipolar disorder; Schizophrenia |
| Quetiapine | Dopaminergic | 5-HT_2A_ and D_2_ antagonist, atypical antipsychotic | Major depressive disorder; Psychotic disorders; bipolar disorder; Schizophrenia |
| Risperidone | Dopaminergic | 5-HT_2A_ and D_2_ antagonist, atypical antipsychotic | Major depressive disorder; Psychotic disorders; bipolar disorder; Schizophrenia |
| Pimavanserin | Dopaminergic | 5-HT_2A_ inverse agonist without D_2_ binding | Parkinson’s disease psychosis |
| Lorcarserin | Dopaminergic | 5-HT_2C_ receptor agonist | Weight loss |
| Alosetron | Dopaminergic | 5-HT_3_ receptor antagonist | Antiemetic |
| Cilansetron | Dopaminergic | 5-HT_3_ receptor antagonist | Antiemetic |
| Dolasetron | Dopaminergic | 5-HT_3_ receptor antagonist | Antiemetic |
| Granisetron | Dopaminergic | 5-HT_3_ receptor antagonist | Antiemetic |
| Ondansetron | Dopaminergic | 5-HT_3_ receptor antagonist | Antiemetic |
| Palonosetron | Dopaminergic | 5-HT_3_ receptor antagonist | Antiemetic |
| Tetrabenazine | Dopaminergic | CNS-active agent  Reversible inhibitor of mono-amine uptake (dopamine depletion) | Hyperkinetic movement disorders such as Huntington’s chorea or Tourette syndrome. |
| Mirtazapine | Dopaminergic | atypical antidepressant | Major depressive disorder  Insomnia |
| Nefazodone | Dopaminergic | atypical antidepressant | Major depressive disorder |
| Trazodone | Dopaminergic | atypical antidepressant | Major depressive disorder; Insomnia |
| Amisulpride | Dopaminergic | D_2_ and D_3_ antagonist | Schizophrenia; Major depressive disorder |
| Amphetamine | Dopaminergic | DAT ligand (interacts with dopamine transporter) | Attention-deficit/hyperactivity disorder; Attention-deficit disorder; Narcolepsy; Obesity |
| Cocaine | Dopaminergic | DAT ligand (interacts with dopamine transporter) | Recreational  Local anesthetic |
| Methamphetamine | Dopaminergic | DAT ligand (interacts with dopamine transporter) | Attention-deficit/hyperactivity disorder; Attention-deficit disorder; Narcolepsy; Obesity |
| Methylphenidate | Dopaminergic | DAT ligand (interacts with dopamine transporter) | Attention-deficit/hyperactivity disorder; Attention-deficit disorder; Narcolepsy; Obesity |
| Bupropion | Dopaminergic | DAT ligand (interacts with dopamine transporter), atypical antidepressants | Major depressive disorder; Smoking cessation |
| Apomorphine | Dopaminergic | dopamine receptor agonist | Parkinson’s disease; Restless legs syndrome |
| Bromocriptine | Dopaminergic | dopamine receptor agonist | Parkinson’s disease; Hyperprolactinemia  Galactorrhea/ Hyperprolactinemia  Acromegaly |
| Cabergoline | Dopaminergic | dopamine receptor agonist | Parkinson’s disease; Galactorrhea/Hyperprolactinemia |
| Pramipexole | Dopaminergic | dopamine receptor agonist | Parkinson’s disease; Restless legs syndrome |
| Ropinirole | Dopaminergic | dopamine receptor agonist | Parkinson’s disease; Restless legs syndrome |
| Rotigotine | Dopaminergic | dopamine receptor agonist | Parkinson’s disease; Restless legs syndrome |
| Clozapine | Dopaminergic | dopamine receptor antagonist, 5-HT_2A_ and D_2_ antagonist | Schizophrenia |
| Cariprazine | Dopaminergic | dopamine receptor antagonist, D_2_ partial agonist | Schizophrenia |
| Brexpiprazole | Dopaminergic | dopamine receptor antagonist, D_2_ partial agonist, atypical antipsychotic | Major depressive disorder; psychotic disorders; bipolar disorder; Schizophrenia |
| Chlorpromazine | Dopaminergic | dopamine receptor antagonist, low-potency D_2_ antagonist | Schizophrenia; psychotic disorders |
| Haloperidol | Dopaminergic | dopamine receptor antagonist, medium- and high-potency D_2_ antagonist | Schizophrenia; psychotic disorders |
| Rasagiline | Dopaminergic | MAO-B inhibitors (reduce oxidative metabolism of dopamine) | Parkinson’s disease |
| Fluphenazine | Dopaminergic | medium- and high-potency D_2_ antagonist | Schizophrenia |
| Loxapine | Dopaminergic | medium- and high-potency D_2_ antagonist | Schizophrenia |
| Perphenazine | Dopaminergic | medium- and high-potency D_2_ antagonist | Schizophrenia |
| Thiothixene | Dopaminergic | medium- and high-potency D_2_ antagonist | Schizophrenia |
| Trifluoperazine | Dopaminergic | medium- and high-potency D_2_ antagonist | Schizophrenia |
| Isocarboxazid | Dopaminergic | monoamine oxidase inhibitor | Major depressive disorder |
| Phenelzine | Dopaminergic | monoamine oxidase inhibitor | Major depressive disorder |
| Tranylcypromine | Dopaminergic | monoamine oxidase inhibitor | Major depressive disorder |
| Selegiline | Dopaminergic | monoamine oxidase inhibitor, MAO-B inhibitors (reduce oxidative metabolism of dopamine) | Major depressive disorder; Parkinson’s disease |
| Flibanserin | Dopaminergic | multifunctional serotonin agonist and antagonist | Sexual dysfunction |
| Ergonovine | Dopaminergic | multiple 5-HT receptor isoforms (ergots) | Postpartum hemorrhage |
| Lysergic acid diethylamide | Dopaminergic | multiple 5-HT receptor isoforms (ergots) | Recreational |
| Methylergonovine | Dopaminergic | multiple 5-HT receptor isoforms (ergots) | Postpartum hemorrhage |
| Methysergide | Dopaminergic | multiple 5-HT receptor isoforms (ergots) | Migraine |
| Desvenlafaxine | Dopaminergic | serotonin-norepinephrine reuptake inhibitor | Generalized anxiety disorder; Major depressive disorder; Attention-deficit/hyperactivity disorder; Autism |
| Duloxetine | Dopaminergic | serotonin-norepinephrine reuptake inhibitor | Stress-induced incontinence  Neuropathic pain |
| Levomilnacipran | Dopaminergic | serotonin-norepinephrine reuptake inhibitor | Generalized anxiety disorder; Major depressive disorder; Attention-deficit/hyperactivity disorder; Autism |
| Milnacipran | Dopaminergic | serotonin-norepinephrine reuptake inhibitor | Generalized anxiety disorder; Major depressive disorder; Attention-deficit/hyperactivity disorder; Autism |
| Venlafaxine | Dopaminergic | serotonin-norepinephrine reuptake inhibitor | Generalized anxiety disorder; Major depressive disorder; Attention-deficit/hyperactivity disorder; Autism |
| Amitriptyline | Dopaminergic | Tricyclic antidepressant | Major depressive disorder  Migraine-prophylaxis  Insomnia |
| Amoxapine | Dopaminergic | Tricyclic antidepressant | Major depressive disorder |
| Clomipramine | Dopaminergic | Tricyclic antidepressant | Major depressive disorder |
| Desipramine | Dopaminergic | Tricyclic antidepressant | Major depressive disorder |
| Doxepin | Dopaminergic | Tricyclic antidepressant | Major depressive disorder; Insomnia |
| Imipramine | Dopaminergic | Tricyclic antidepressant | Major depressive disorder |
| Maprotiline | Dopaminergic | Tricyclic antidepressant | Major depressive disorder |
| Nortriptyline | Dopaminergic | Tricyclic antidepressant | Major depressive disorder |
| Protriptyline | Dopaminergic | Tricyclic antidepressant | Major depressive disorder |
| Trimipramine | Dopaminergic | Tricyclic antidepressant | Major depressive disorder |
| Abbreviations: Ach: acetylcholine. CNS: central nervous system. NMDA: N-methyl-D-aspartate. 5-HT: 5-hydroxytryptamine. DAT: dopamine transporter. MAO-B: Monoamine oxidase B  *Common uses are revised based on FDA Approval History to also reflect common uses in real life practice. | | | |
